# The Carbonic Anhydrase IX inhibitor SLC-0111 as emerging agent against the mesenchymal stem cell-derived pro-survival effects on melanoma cells

**DOI:** 10.1080/14756366.2020.1764549

**Published:** 2020-05-12

**Authors:** Silvia Peppicelli, Elena Andreucci, Jessica Ruzzolini, Francesca Bianchini, Chiara Nediani, Claudiu T. Supuran, Lido Calorini

**Affiliations:** a Department of Clinical and Experimental Biomedical Sciences “Mario Serio”, Section of Experimental Pathology and Oncology, University of Florence, Florence, Italy; b Department of NEUROFARBA, University of Florence, Florence, Italy; c Center of Excellence for Research, Transfer and High Education, DenoTHE University of Florence, Florence, Italy

**Keywords:** SLC-0111, CAIX inhibitor, MSC, melanoma

## Abstract

Mesenchymal stem cells (MSC) take part to solid tumour-associated stroma and critically influence progression of malignancy. Our study represents a striking example of melanoma progression to a more malignant and resistant phenotype promoted by MSC and the possibility to contrast this diabolic liaison using CAIX inhibitors. In particular, we demonstrated that melanoma cells exposed to a MSC-conditioned medium switch to a more malignant phenotype, characterised by resistance to programmed cell death and endowed with an epithelial-to-mesenchymal transition and stem cell characteristics. These effects were reversed abrogating MSC CAIX activity using SLC-0111, a CAIX inhibitor. Moreover, the acquisition by melanoma cells of a Vemurafenib-resistant phenotype upon MSC-conditioned medium exposure was removed when MSC were treated with SLC-0111. Therefore, MSC may profoundly reprogramme melanoma cells towards a wide resistant phenotype through CAIX involvement, as the use of SLC-0111 is able to contrast the development of this highly risky adaptation for disease progression.

## 1. Introduction

Malignant cancer cells proliferate and expand in an aggressive manner into the host adjacent tissues, penetrate the vasculature and, possibly, colonise new organ sites. Cancer cells express an autonomous growth ability which may take the advantage to be stimulated by the host normal cells characterising to so-called tumour microenvironment (TME)^1^^,^^2^. It is well known that growth of cancer cells is not only dependent by the genetic and epigenetic changes accumulated by cancer cells during their progression towards a more malignant phenotype, but also depends on tumour stroma, humoral and cellular components of their macro- and micro-environment. Indeed, the composition of tumour stroma is quite similar to that of normal tissues, and why the host provides a tumour tissue with the stroma it needs to grow and disseminate is still not well understood^3^. In the majority of tumours, TME is also characterised by hypoxia and acidosis, aspects that strongly influence the biology of cancer cells^4^. Among the several inflammatory cells of TME, most common solid tumours contain a large number of resting and activated fibroblasts, which are the principal directors in extracellular matrix (ECM) homeostasis, either producing fibrillary ECM or generating ECM-degrading proteases, such as matrix metalloproteinases^5^. Fibroblasts are also capable of secreting various growth factors which sustain cancer cells, and, at the same time, cancer cells sustain fibroblasts activation leading to the induction of a critical cell population indicated as “cancer-associated fibroblasts” (CAF) (also referred myofibroblasts), which express an even higher secretory activity. Some years ago, Dvorak suggested that tumours behaved as wounds that do not heal, e.g. tumours induce the stroma they required for growth co-opting the wound healing response process, and disclosing that fibroblasts of the tumour stroma closely resemble fibroblasts associated with wound healing^6^. Indeed, CAF are, although not always, characterised by the expression of α-smooth-muscle actin and their most important inducer is the transforming growth factor (TGF)-β1, which also mediates wound healing fibroblasts activation and organ fibrosis. CAF in addition to secrete growth factor for cancer cells, are a source of ECM-degrading proteases which assist cancer cells to overcome host anatomical barriers and angiogenic growth factors that support tumoral angiogenesis. In particular, CAF release MMP3 (matrix-metalloprotease 3), which upon the cleavage of E-cadherin extracellular domain, promotes epithelial-to-mesenchymal transition (EMT) of cancer cells. EMT is a crucial reprogramming phenotype associated with easing cancer cells migratory ability, apoptosis resistance and stemness. While investigating prostate carcinoma cell/CAF crosstalk it has been found that tumoral interleukin-6-activated fibroblasts promote, in cancer cells, an EMT phenotype endowed with stem cell markers^7^.

Understanding the origin of CAF is complex, in view of the heterogeneity of mesenchymal cell population itself. Indeed, CAF may derive from local fibroblasts, from the trans-differentiation of several cell types of cells, including endothelial cells, and from bone-marrow-derived mesenchymal stem cells (MSC). Recent contributions proposed that MSC is one of the largest population recruited into the primary tumours^8^, and that TGF-β1, not only, plays a crucial role in MSC attraction towards prostate cancer, but also activates MSC to CAF-like cells^9^. Currently it is well established that MSC have a central effects on cancer cells survival, angiogenesis, EMT and in the promotion of primary and metastatic lesions growth^10^.

We have demonstrated that MSC-conditioned medium (MSC-CM) induces in melanoma cells an EMT profile and that TGF-β silencing, in MSC, was found to abrogate ability of MSC to promote EMT in melanoma cells^11^. We also found that MSC grown in a low pH (LpH-MSC) medium stimulate melanoma xenografts growth more efficently than MSC grown in a standard pH medium. Melanoma cells exposed to a LpH-MSC medium express an EMT phenotype associated with a forced glycolytic pathway^12^. These latter findings clearly show how stromal cells and their physical environment, e.g. acidosis of TME, may cooperate in tumour progression.

Cancer cells successfully adapt to environmental stresses, changing their molecular asset and their phenotype. The level of adaptation is the consequence of cancer cell exposure to the complexity of the tumour micro-environmental stimuli, which depend on the presence of pro-inflammatory cells, and on the hypoxia and/or acidosis of the surrounding region in which cancer cell might be located at that moment. One of the crucial and consistent markers of tumour cells adaptation to local stresses is represented by the expression of high level of carbonic anhydrase (CA) IX. CAIX is a metalloenzyme that catalyses the reversible formation of HCO_3_^−^ and H^+^ ions from H_2_O and CO_2._ CAIX expression is promoted by hypoxia inducible factors 1α (HIF-1α) in the hypoxic regions within the tumour mass^13^^,^^14^ and also by extracellular acidic microenvironment via HIF-1α-independent mechanisms^15^^,^^16^. CAIX expression is associated with migration, invasion and stemness of cancer cells^17^. We have previously demonstrated an increased CAIX expression in melanoma, breast and colorectal cancer cells when transiently and chronically exposed to an extracellular acidic microenvironment (pH 6.7 ± 0.1)^18^. Extracellular acidosis represents a critical characteristic of most solid tumours, often associated with aggressive phenotypes and resistance to therapies^19^. Moreover, we demonstrated that SLC-0111, a CAIX inhibitor, is able not only to prevent such CAIX increased expression, but also to selectively induce the apoptotic programme in A375-M6 melanoma cells, MCF7 breast cancer cells and HCT116 colorectal cancer cells transiently and chronically exposed to extracellular acidosis. It has to be noted that any cytotoxic effect was found in the population maintained under standard pH condition (pH 7.4 ± 0.1) after SLC-0111 treatment^18^.

In this study, we have chosen the human melanoma system to investigate the hypothesis that SLC-0111, a novel ureido-substituted benzenesulfonamide CAIX inhibitor, may abrogate the pro-survival properties induced in tumour cells by MSC-conditioned medium. We found that the SLC-0111 administration to MSC is able to inhibit in melanoma cells not only the induced proliferation, resistance to apoptosis and anoikis, but also the stimulation of EMT and stemness phenotype. Moreover we showed for the first time that in melanoma cells MSC induce a Vemurafenib-resistant phenotype and that this effect was abrogated when MSC were treated with the SLC-0111 CAIX inhibitor.

## 2. Material and methods

### 2.1. Cell cultures

Human melanoma cell line A375-M6, isolated in our laboratory as previously described^20^, was maintained in DMEM 4,5 g/L glucose and 2 mM L-glutamine supplemented with 10% foetal bovine serum (Euroclone, Milan, Italy). Mesenchymal Stem Cells (MSC) obtained from bone marrow aspirates of donors, which signed informed consent, were expanded in Dulbecco’s modified Eagle’s medium with low glucose (DMEM 1000; Gibco, Life Technologies, MB, Italy), supplemented with foetal bovine serum 20% and incubated at 37 °C in humidified atmosphere containing 95% air and 5% CO_2_ according with Peppicelli *et al*.^12^. Cells were harvested from subconfluent cultures by incubation with a trypsin-EDTA solution (Life Technologies), and propagated every 3 days. Viability of the cells was determined by trypan blue exclusion test. Cultures were periodically monitored for mycoplasma contamination using Chen’s fluorochrome test^21^.

### 2.2. Cell treatments

CAIX inhibitor SLC-0111, developed in the laboratory of Professor Claudiu T. Supuran (NEUROFARBA Department, University of Florence, Italy) and previously described^18^, was used at 100 µM dose to treat MSC for 24 h.

Etoposide (MedChemExpress, Sollentuna, Sweden) 100 µM was administrated to melanoma cells for 24 h to induce apoptosis.

Vemurafenib (MedChemExpress) 1 mM was used to treat for 24 h A375-M6 melanoma cells grown in standard medium or in MSC conditioned medium.

### 2.3. Flow cytometry

MSC were harvested by using Accutase (Euroclone), collected in flow cytometer tubes (2 × 10^5^ cells/tube), and incubated 1 h at 4 °C with anti-CAIX antibody (Merck Millipore), as previously described^18^. Cells were washed in PBS and incubated 1 h in the dark at 4 °C with anti-mouse antibody conjugated with Alexa Fluor 488 (Invitrogen). Samples were washed in PBS and re-suspended in 500 µL PBS to proceed with the analysis at BD FACS Canto (BD Biosciences, Franklin Lakes, New Jersey, USA). The flow cytometer was calibrated using cells incubated with secondary antibody only.

### 2.4. Preparation of conditioned medium by MSC

MSC were seeded at density of 1 × 10^5^ cell/ml in complete media in T-25 tissue culture flasks and grown to 70% of confluence as previously described^12^. Cells were treated with SLC-0111 for 24 h and then washed twice with PBS and incubated in DMEM 4500, 2% FCS medium for 24 h. Conditioned media were collected and filtered with a 0.22 mm filter and tumour cells were incubated in MSC conditioned media for 24 h.

### 2.5. Cell death evaluation

Cell death was determined by flow cytometer analysis using Annexin V FITC/APC or FITC-conjugated (Immunotools GmbH, Germany) and PI (Sigma-Aldrich) as described in^22^. Briefly, cells treated with Etoposide were harvested with Accutase (Euroclone), collected in flow cytometer tubes (1 × 10^5^ cells/tube), washed in PBS and incubated 15 min at 4 °C in the dark with 100 µL Annexin Binding buffer (100 mM HEPES, 140 mM NaCl, 25 mM CaCl_2_, pH 7.4), 1 µL of 100 µg/ml PI working solution, and 5 µL Annexin V FITC/PI-conjugated. Each sample was added with Annexin Binding Buffer to reach 500 µL volume/tube. Samples were then analysed at BD FACS Canto (BD Biosciences).

### 2.6. Western Blot analysis

Cells were lysed in RIPA buffer (Merck Millipore, Vimodrone, MI, Italy) containing PMSF (Sigma-Aldrich, Saint Louis, Missouri, USA), sodium orthovanadate (Sigma-Aldrich), and protease inhibitor cocktail (Calbiochem, San Diego, CA, USA), sonicated and centrifuged 15 min at 14,000 rpm at 4 °C. Equal amounts of protein were separated on Bolt^®^ Bis-Tris Plus gels, 4–12% precast polyacrylamide gels (Life Technologies, Monza, Italy). Fractionated proteins were transferred to a PVDF membrane using the iBlot 2 System (Life Technologies). Following 1 h blocking with Odyssey blocking buffer (Dasit Science, Cornaredo, MI, Italy), membrane was probed overnight at 4 °C with mouse anti -N-Cadherin and mouse anti-E-Cadherin (1:1000, DAKO, Glostrup, Denmark); rabbit anti-EGFR, (1:500 Cell Signalling Technology, Danvers, MA, USA); rabbit anti-PDGFR, rabbit anti p-p70S6k, rabbit anti-pERK, rabbit anti-ERK (1:1000, Cell Signalling Technology, Danvers, MA, USA), and 1 h at room temperature with goat anti-rabbit IgG Alexa Flour 750 antibody (Invitrogen, Monza, Italy). Membrane was visualised by the Odyssey Infra-red Imaging System (LI-COR^®^ Bioscience, Lincoln, Nebraska USA). Anti α-tubulin antibody (1:2000, Sigma-Aldrich) was used to assess equal amount of protein loaded in each lane.

### 2.7. Colony formation assay

2 × 10^2^ A375-M6 cells were seeded in 6-well plate and grown for 12 days in standard medium or in medium conditioned by MSC. Developed colonies were counted upon 20 min-fixation in 4% paraformaldehyde at 4 °C and 30 min-staining with Crystal violet solution at room temperature.

### 2.8. Invasion assay

Invasiveness of A375-M6 melanoma cells grown in media conditioned by MSC was determined *in vitro* on Matrigel (BD Biosciences) -precoated polycarbonate filters, with 8 µm pore size, 6.5 mm diameter, 12.5 µg Matrigel/filter, mounted in Boyden’s chambers as previously described^20^. 1,5 × 10^5^ cells (200 µL), were seeded in the upper compartment and incubated for 6 h at 37 °C in 10% CO_2_ in air. In the lower chamber, complete medium was added as chemo attractant. After incubation, the inserts were removed and the non invading cells on the upper surface were wiped off mechanically with a cotton swab and the membranes were fixed overnight in ice-cold methanol. Cells on the lower side of the membranes were then stained using the Diff-Quick kit (BD Biosciences) and photographs of randomly chosen fields are taken.

### 2.9. Rna isolation and quantitative PCR (qPCR)

Total RNA was extracted from cells by using TRI Reagent (Sigma). The amount and purity of RNA were determined spectrophotometrically. cDNA synthesis was obtained by incubating 2 µg of total RNA with 4 U/µL of M-MLV reverse transcriptase (Promega, San Luis Obispo, California) according to the manufacturer’s instructions.

Quantitative real time PCR (qPCR) was performed using the GoTaq^®^ Probe Systems (Promega). The qPCR analysis was carried out in triplicate using an Applied Biosystems 7500 Sequence Detector with the default PCR setting: 40 cycles of 95° for 15 s and 60 °C for 60 s. mRNA was quantified with the ΔΔCt method as described^23^. mRNA levels were normalised to β-2 microglobulin and β-actin as endogenous controls. Primer sequences are reported in Table 1.

**Table 1.** Primer sequences for PCR.

Gene	Primer Fw	Primer Rv
β-2 microglobulin	5′-GCCGTGTGAACCATGTGACT-3′	5′-GCTTACATGTCTCGATCCCACTT-3′
β-actin	5′-TCGAGCCATAAAAGGCAACT-3′	5′-CTTCCTCAATCTCGCTCTCG-3′
KFL4	5′-GCAGCCACCTGGCGAGTCTG-3′	5′-CCGCCAGCGGTTATTCGGGG-3′
c-Myc	5′-AATGAAAAGGCCCCCAAGGTAGTTAT-3′	5′-GTCGTTTCCGCAACAAGTCCTCTTC-3′
NANOG	5′-ACCTTGGCTGCCGTCTCTGG-3′	5′-AGCAAAGCCTCCCAATCCCAAACA-3′
OCT3/4	5′-TTTTGGTACCCCAGGCTATG-3′	5′-GCAGGCACCTCAGTTTGAAT-3′
SOX2	5′-GAGCTTTGCAGGAAGTTTGC-3′	5′-GCAAGAAGCCTCTCCTGAA-3′

### 2.10. Anoikis assay

In order to simulate anchorage-independent cell growth condition, we performed the rocking test: cells grown in MSC-conditioned media were placed in tubes and they were leave to rock on the Mini Rocker Shaker (Biosan), at room temperature for 48 h in Dulbecco’s Mem Nutrient mix F12 (DMEM/F12-HEPES EuroClone, MI, Italy). After treatment, cells were seeded in 100 mm plates (5000 cells/dishes, in triplicate) and incubated for 12 days.

### 2.11. Melanosphere formation assay

A375-M6 cells grown in standard or MSC-conditioned media were counted. Single cancer cells were plated at 150 cells/cm^2^ on a low-attachment 100 mm plate in a Dulbecco’s modified Eagle’s medium/F12 supplemented with N2 media, 5 g/ml insulin, 20 ng/ml fibroblast GF-2, and 20 ng/ml epidermal GF (all from Thermo Fisher Scientific). Cells were grown under these conditions for 10–15 days and formed non adherent spheres, which were photographed.

### 2.12. Statistical analysis

The experiments were performed at least three times for a reliable application of statistics. Statistical analysis was performed with GraphPad Prism software. Values are presented as mean ± SD. N value represents the number of biological replicates. One- and Two-way ANOVA were used to evaluate the statistical significance.

## 3. Results

### 3.1. The CAIX SLC-0111 inhibitor reverts MSC reprogramming of melanoma cells towards a more malignant phenotype

Considering our previous works on the tumour promoting effect of MSC, such as resistance to apoptosis and epithelial-to-mesenchymal transition (EMT)^11^^,^^12^, and supporting by the high expression of CAIX in MSC (see Supplementary, Figure S1), we decided to verify whether the CAIX inhibitor SLC-0111 might be able to abolish the MSC tumour promoting effects.

Thus, we exposed A375-M6 melanoma cells to a medium conditioned by MSC (cmMSC) or MSC treated for 24 h with 100 µM SLC-0111 (cmMSC-SLC-0111). Melanoma cells were evaluated for their proliferation, resistance to apoptosis and EMT markers.

**Figure 1.**
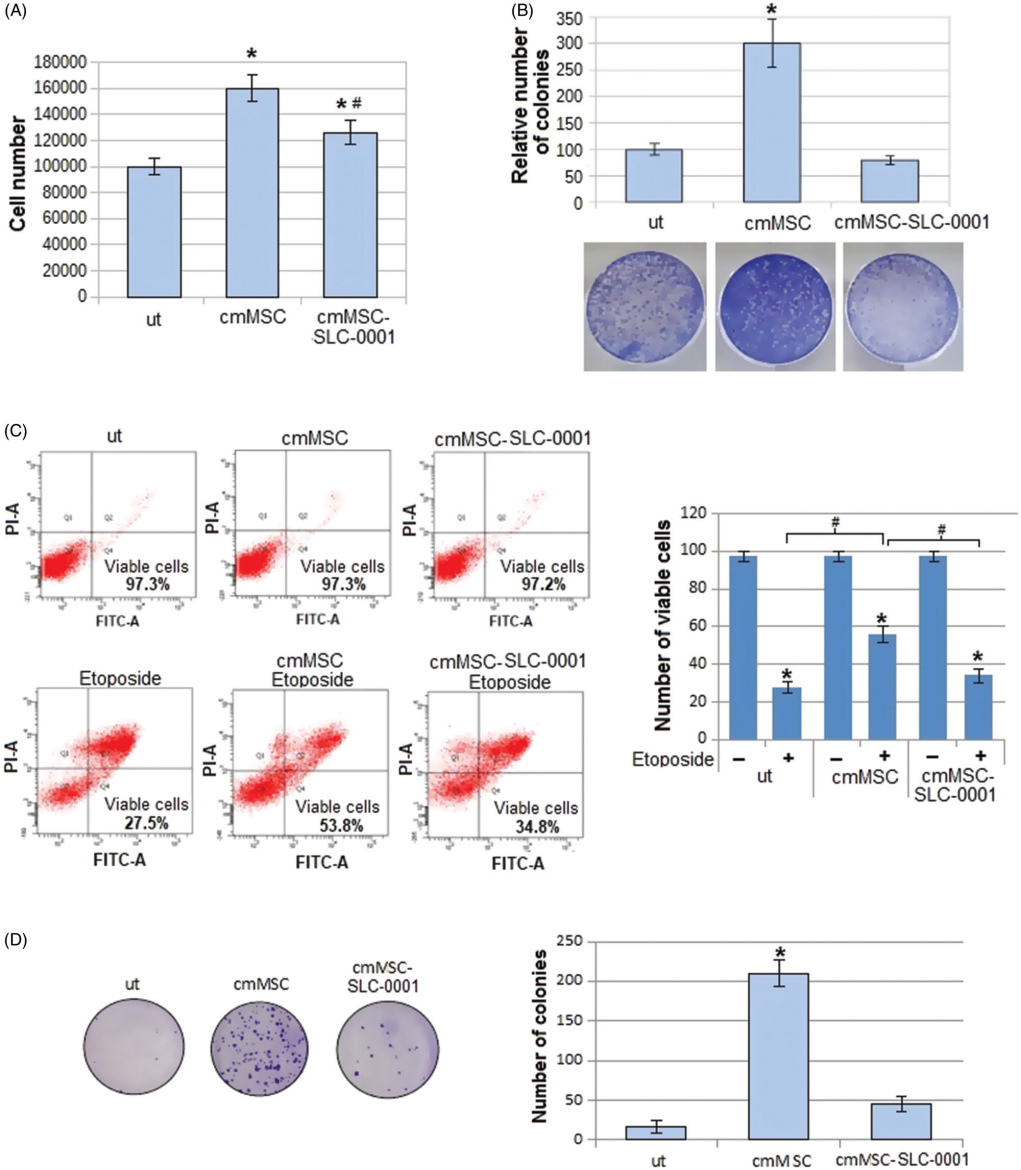
Effect of CAIX inhibitor administration to MSC, on proliferation and apoptosis resistance of A375-M6 grown in MSC-conditioned media. (A) Number of cells and (B) colonies formed by A375-M6 melanoma cells grown in standard medium (untreated cells, ut), in medium conditioned by MSC (cmMSC) or by MSC treated with SLC-0111 100 μM (cmMSC-SLC-0111). (C) Representative plots of Annexin V/PI assay of A375-M6 cmMSC or cmMSC-SLC-0111 treated with Etoposide 100 μM to induce apoptosis. Cumulative results from 3 experiments are represented on the right. (D) Representative images (left) of clonogenic efficacy of A375-M6 cmMSC and cmMSC-SLC-0111 melanoma cells after exposure to 48 h of rocking condition. After treatment, cells were seeded in 100 mm plates (5000 cells/dishes, in triplicate) and incubated for 12 days. On the right the quantification of cloning efficiency expressed as number of colonies. Data represent the mean ± SEM of at least three independent experiments.*p < .05 compared with control cells. ^#^*p* < .05.

We found that the promotion of the proliferation rate, evaluated either counting number of cells (Figure 1(A)) and colonies (Figure 1(B)), due to the MSC-conditioned medium, was significantly reduced when medium was collected by MSC-treated with the CAIX inhibitor (Figure 1(A,B)).

Along cell proliferation and viability, we tested the ability of melanoma cells to resist to apoptosis induced by the pro-apoptotic drug Etoposide. As shown in Figure 1(C), cell viability reduces by 70% after Etoposide treatment when melanoma cells are grown in standard medium, whereas cell viability reduces only by 44% when melanoma cells are grown in cmMSC. Apoptosis resistance induced by MSC-conditioned medium instead decreased (the percentage of dead cells goes from 44% to 63%) when MSC were pre-treated with the CAIX inhibitor SLC-0111 (Figure 1(C)).

A pro-survival reprogramming elicited by MSC, prompted us to investigate whether MSC-conditioned media and treatment with SLC-0111 may have any effect on the *anoikis* resistance of melanoma cells, a programmed cell death resistance occurring in cancer cells upon detachment from extracellular matrix. Cancer cells need to express *anoikis* resistance when they spread and gain the circulatory vessels to colonise distant organs, e.g. *anoikis* resistance is of a real importance for cancer dissemination and its understanding is or primary importance to identify possible new therapeutic strategies.

To do that, we tested *anoikis* resistance using a rocking procedure as in our previous work^24^. Melanoma cells grown in MSC-conditioned medium were suspended in free growth factor media and placed in sterile non-adhesive 50 ml-tubes fixed on a Mini rocker platform shaker. Time of treatment at a speed of 30 cycles/min was 48 h, at room temperature. At the end of treatment, cells were collected and their cloning efficiency determined. As reported in Figure 1(D), we found that cmMSC melanoma cells express a high capacity to give rise cell clones, and this ability is reduced when cells are exposed to a medium conditioned by MSC treated with SLC-0111, disclosing an important role of CAIX on *anoikis* resistance.

**Figure 2.**
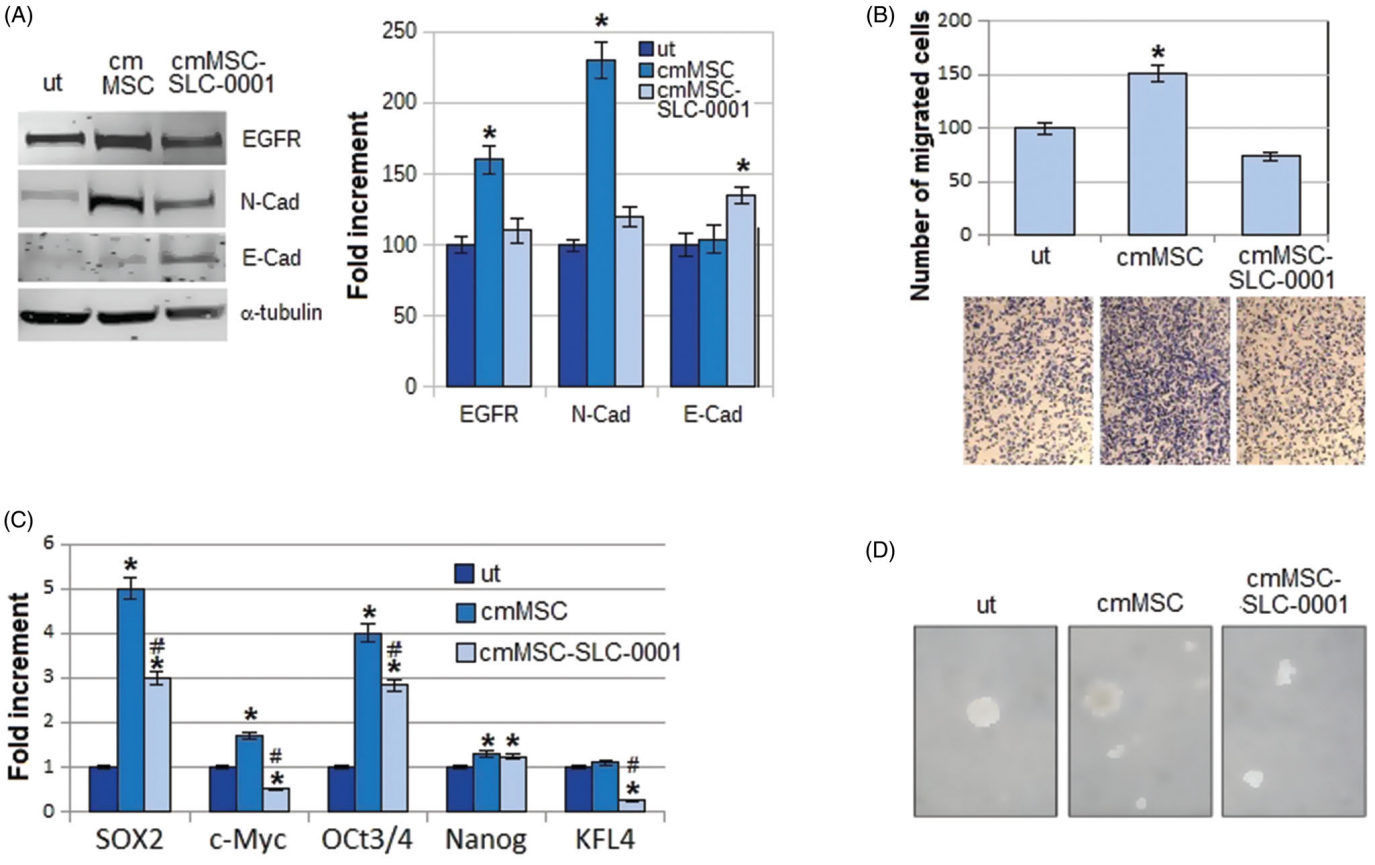
Effect of SLC-0111 administration to MSC on melanoma EMT induced by MSC-conditioned medium. (A) Representative images of western blot for EGFR, N-cadherin, E-Cadherin and *a*-tubulin of A375-M6 melanoma cells grown in standard condition (untreated cells, ut) or in medium conditioned by MSC (cm MSC) or by MSC treated with SLC-0111 (cmMSC-SLC-0111), and (right) densitometry graph of protein expression. (B) Representative images (below) of invasiveness of melanoma cells grown in media conditioned by MSC and quantitative analysis (on top) of the number of cells that migrated through Matrigel. (C) qPCR analysis of the stemness markers SOX2, c-Myc, OCT3/4, Nanog and KFL4 of A375-M6 melanoma cells grown in cmMSC or cmMSC-SLC-0111. (D) Representative images of tumour-spheres formation assay of A375-M6 grown in cmMSC or cmMSC-SLC-0111. Data represent the mean ± SEM of at least three independent experiments.*p < .05 compared with control cells. ^#^*p* < .05 compared with A375-M6 cmMSC.

Overall, either apoptosis or *anoikis* resistance expressed by melanoma cells upon their exposure to MSC media and abrogated by the CAIX SLC-0111 inhibitor suggested to verify whether the EMT programme promoted in melanoma cells by MSC might be inhibited, being the EMT a driver of both resistant conditions. We found that melanoma N-Cadherin expression, induced by MSC-conditioned medium, is reduced when MSC are treated with the SLC-0111, whereas E-Cadherin expression is increased, suggesting the ability of this drug to block the MSC-elicited EMT programme (Figure 2(A)). We also evaluated the expression of EGFR, a well-known regulator of EMT and drug resistance. It is known that the pro-survival activities associated with apoptosis and *anoikis* resistance are effective barriers against an effective chemotherapy. We found that EGFR induction due to the MSC-conditioned medium was reduced when MSC were treated with the CAIX inhibitor (Figure 2(A)). As an additional character of EMT undergoing cancer cells, we tested the ability of melanoma cells to invade through Matrigel-coated filters, and we observed that the higher invasiveness detected in cmMSC A375-M6, was significantly reduced in cmMSC-SLC-0111 cells, confirming the ability of this drug to inhibit all characters of EMT induced by MSC.

To extend our study on the pro-survival effect of MSC possibly inhibited by the SLC-0111 CAIX inhibitor, we evaluated whether this inhibitor might have a sort of effect on some stem cell-like characteristics possibly promoted by MSC. Figure 2(B) shows that medium conditioned by MSC induces the expression of stemness markers, e.g. SOX-2, c-Myc, OCT 3/4, NANOG, and SLC-0111 is able to prevent this enhancement. Moreover, Figure 2(C), shows that SLC-0111 is efficient in inhibiting the *in vitro* sphere formation induced by cm MSC, an additional assay to reveal stemness in cancer cells.

On the whole, MSC represent a real promoter of melanoma malignancy and CAIX plays a central role in this reprogramming event.

### 3.2. The CAIX inhibitor SLC-0111 reverts the MSC-elicited Vemurafenib resistance in melanoma cells inhibiting mTOR pathway

As described in our previous papers^19^^,^^22^, tumour microenvironmental characteristics, such as low pH, participate to promote drug resistance, included Vemurafenib resistance, in BRAF^V600E^ melanoma cells.

**Figure 3.**
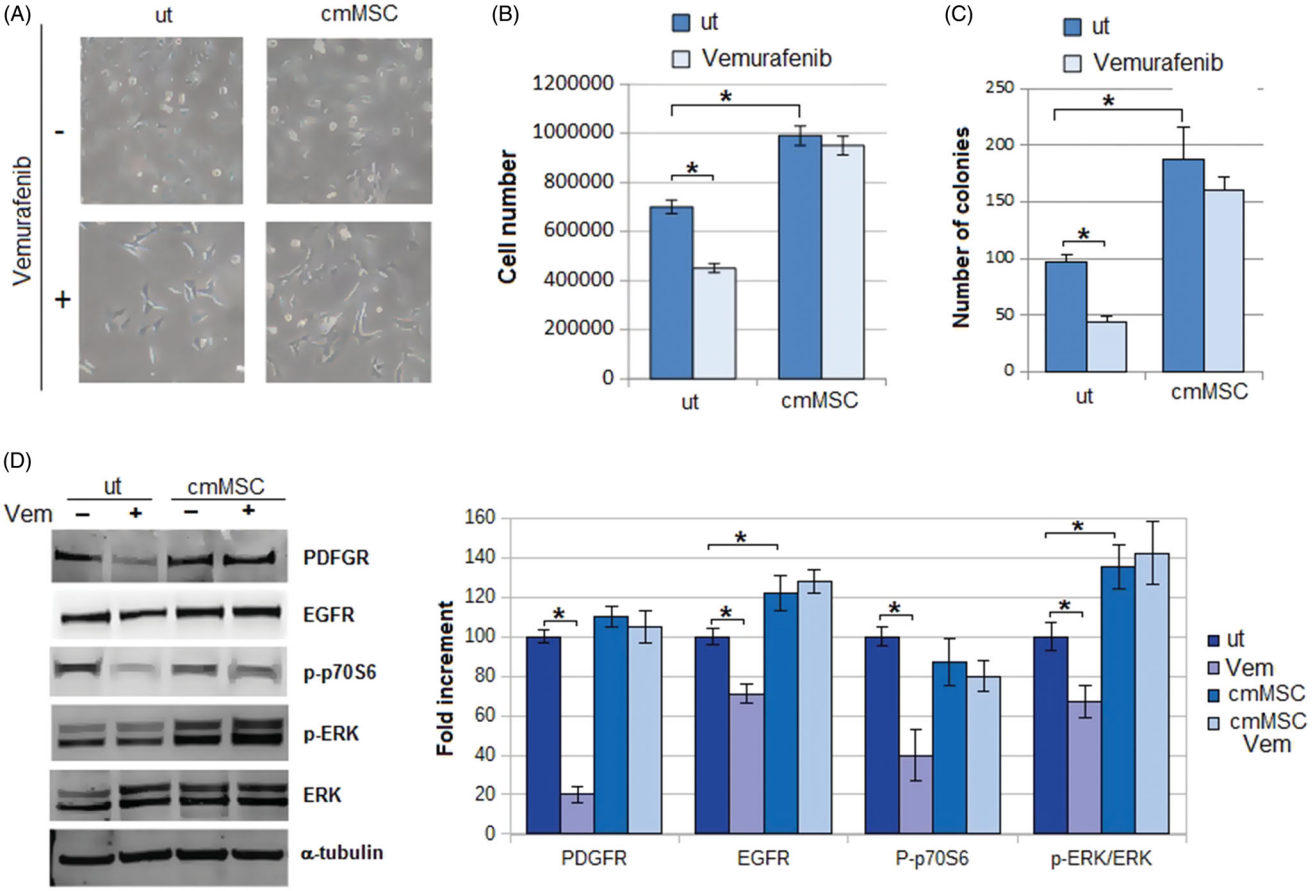
Effects of Vemurafenib addition to A375M6 melanoma cells grown in media conditioned by MSC. (A) Cell morphology, (B) cell number and (C) number of colonies of A375-M6 grown for 24 h in standard medium (untreated cells, ut) or in medium conditioned by MSC. (D) Western blot analysis of p-ERK, ERK, p-p70S6k, PDGFR and PDGFR after 24 h of treatment with Vemurafenib 1 mM. Each band of western blot was quantified by densitometric analysis and the corresponding histogram was constructed as relative to α-tubulin. The corresponding histogram of p-ERK was constructed as relative to ERK and α-tubulin. Representative Western blot panels on the left. Values presented are mean ± SEM of three independent experiments *p < .05 compared with control cells.

We first investigated whether MSC may favour a BRAF inhibitor resistance. A375-M6 melanoma cells were grown for 24 h in medium conditioned by MSC in the presence or absence of 1 mM Vemurafenib. As shown in Figure 3, control cells treated with Vemurafenib displayed an elongated morphology (Figure 3(A)), a reduced proliferation (Figure 3(B)), and a low efficiency to form colonies (Figure 3(C)). When A375M6 were grown in MSC-conditioned medium, Vemurafenib treatment did not change neither cell morphology (Figure 3(A)), nor cell proliferation (Figure 3(B)), nor the ability to give colonies (Figure 3(C)), signifying a clear acquisition of Vemurafenib resistance. This is confirmed by the findings that, melanoma cells grown in MSC-conditioned medium, unlike control cells, do not express a reduction of PDGFR, EGFR and p-ERK after Vemurafenib treatment (Figure 3(D)), well characterised markers of melanoma resistance to Vemurafenib^25^. On the contrary, treatment with Vemurafenib reduced the expression of p-p70S6k in control cells but not in cmMSC melanoma cells, confirming the resistance of A375-M6 cmMSC to the BRAF inhibitor (Figure 3(D)). p-p70S6k, is a well characterised downstream effector of the p-AKT/mTOR pathway, which we and others found responsible for the Vemurafenib resistance of melanoma cells^22^^,^^26^^,^^27^.

**Figure 4.**
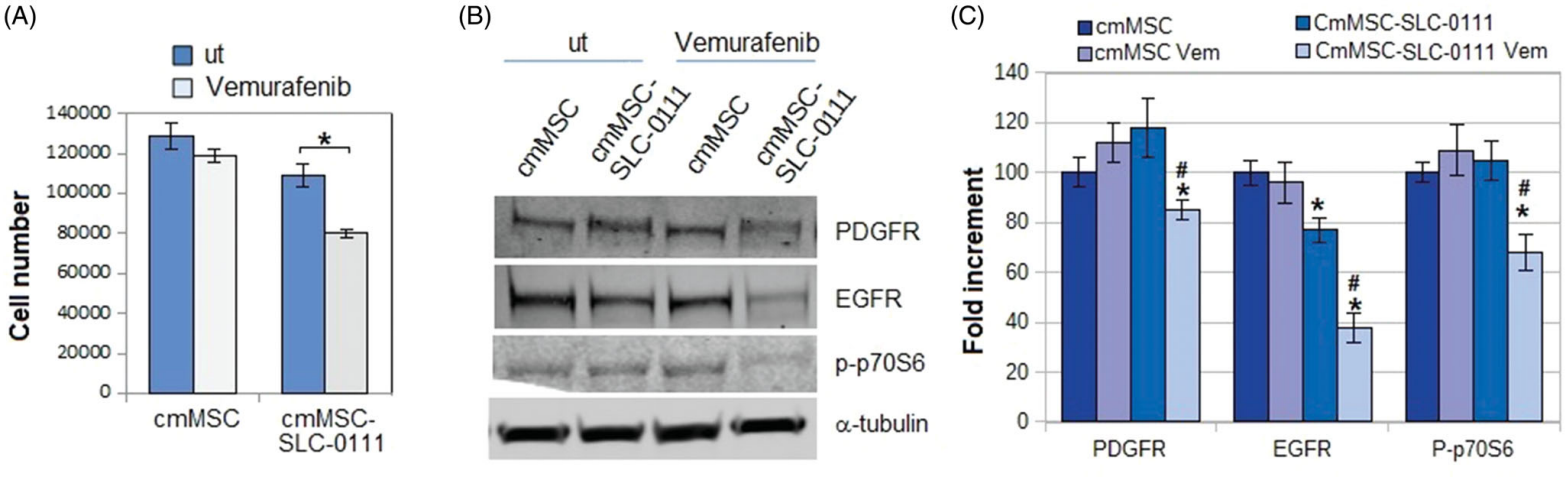
Effect of SLC-0111 administration to MSC on Vemurafenib resistance induced in A375-M6 by MSC-conditioned medium. (A) Number of cells and (B) Western blot analysis of PDGFR, EGFR, p-p70S6k expression in A375-M6 grown in medium conditioned by MSC (cmMSC) or by MSC treated with SLC-0111 (cmMSC-SLC-0111), after Vemurafenib administration. Values presented are mean ± SEM of three independent experiments *p < .05 compared with untreated cells. ^#^*p* < .05 compared with A375M6 cmMSC-SLC-0111.

At this point, we wondered whether the treatment of MSC with SLC-0111 could be able to reverse the A375-M6 resistant phenotype induced by MSC-conditioned medium. Figure 4(A) shows that SLC-0111 re-sensitizes A375-M6cmMSC to the Vemurafenib activity of reducing proliferation, PDGFR, EGFR and p-p70S6k expression (Figure 4(B)).

Therefore, our results suggest that SLC-0111 may add Vemurafenib activity to overcome MSC-promoted drug resistance.

## 4. Discussion and conclusions

It is now well accepted that invasive and metastatic phenotype of cancer cells is not only related to genomic instability characterising certain subpopulations of cancer cells, but also depends from signals originated by tumour-associated stroma, e.g. regional hypoxia and acidosis, and the associated mesenchymal cells. MSC represent one of the most frequent cell populations found in tumour-associated stroma of solid tumours^28^ but their recruitment to tumours and their ability to influence growth, angiogenesis, invasiveness, metastatic dissemination and resistance to therapy, although extensively investigated, are still debated^10^. Studying breast carcinoma cells, Karnoub *et al*.^29^ found that MSC promote in cancer cells a reversible phenotype endowed with an enhanced invasive and metastatic properties, dependent on MSC CCL5 chemokine acting on tumoral receptor CCR5. Along this finding, it was reported that both sulphonamide and coumarin CA inhibitors reduced in a significant manner both tumour growth and metastases of CAIX-positive 4T1 mammary cancer cell xenografts^30^. CAIX is highly expressed, in addition to cancer cells, on normal stroma-associated cells, then is potently induced by hypoxia^31^ and also by acidosis^16^^,^^18^ indicating its wide distribution along the several changes of tumour-associated stroma^15^.

Here, we have reported the efficacy of the SLC-0111 CAIX inhibitor on the abrogation of most the pro-survival effects of MSC, including a first time disclosed promotion of *anoikis* resistance and stem cell markers. In addition, the efficacy of SLC-0111 CAIX inhibitor on the various tumour histotypes prompted its use in a clinical trial (now in Phase II)^32^, and could support us for a positive result^33^.

**Figure 5.**
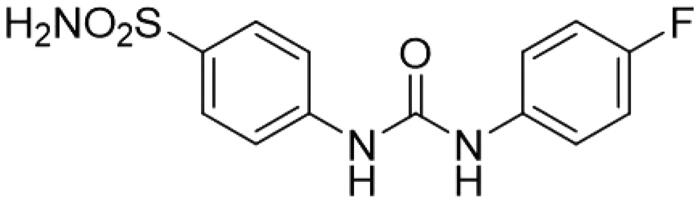
Chemical structure of SLC-0111.

Indeed, SLC-0111 is an ureido sulphonamide derivative (Figure 5) showing CA IX/XII selective inhibitory power^30^. Its inhibition constants against CA IX and XII are of 45 and 4.5 nM, respectively, whereas it is a week, micromolar inhibitor of off-target isoforms such as CA I and II^30^.

The proliferation parameters of melanoma cells, cell number and colonies, that were stimulated by MSC-conditioned medium, recovered the levels of control cells grown in a standard medium when MSC were treated with the SLC-0111. Moreover, while melanoma cells exposed to Etoposide, which targets DNA topoisomerase II activity, undergo apoptosis in almost 70% of cell population, the treatment with a MSC medium reduced the Etoposide lethality on melanoma cells to almost 40%. Interestingly, when we exposed melanoma cells to SLC-0111 CAIX inhibitor, protection of MSC medium ceases and the lethality returns to the control values.

In order to progress to the vertical growth phase and metastatic spread, melanoma cells need to get a resistance to survive also losing contact with the extracellular matrix, the so-called *anoikis* resistance. This activity is mediated by integrins, through survival cell signalling of RAF–MEK–ERK1/2 and PI-3 kinase–AKT^34^. It was found by Chafe *et al*.^35^ that CAIX participates to the development of a pre-metastatic niche one of the critical effect of circulating tumour cells endowed with a strong *anoikis* resistance. Here, we demonstrate that MSC medium stimulated *anoikis* resistance in melanoma cells grown in a rolling condition, disclosing how wide is the pro-survival promotion activity of MSC. Our results showed also that the SLC-0111 was able to abolish the pro-*anoikis* resistance effect of MSC-conditioned medium.

Most of the survival properties expressed by cancer cells, including apoptosis and *anoikis* resistance, are tightly regulated by a reprogrammed phenotype driven by EMT. EMT is involved in several steps of metastatic cascade, such as escaping from the tumour mass, entry into blood vessels, resistance in the circulation, penetration and surviving in a new site. Although melanocytes are derived from cells of neural crest, EMT is a well-documented phenomenon also in melanoma progression^36^^,^^37^. In fact, MSC medium significantly stimulates N-cadherin expression and invasion through a Matrigel barrier of melanoma cells: both of these features are key makers of EMT. The SLC-0111 CAIX inhibitor abrogated the ability of MSC medium to promote EMT, reducing both invasiveness and N-cadherin expression, which was counterbalanced by an increase in E-cadherin expression. In addition, Shin *et al*.^38^ suggested that CAIX overexpression reduces adhesiveness and promotes cell migration by Rho-GTPase signal transduction pathway in human cervical cancer cells.

Recent studies revealed that epithelial cancer cells undergoing EMT, also acquire a stem cell-like phenotype. Further, Voon *et al*.^39^ referred that the EGFR/Ras pathway is required for the generation of a stem cell phenotype in gastric cancer cells, a process associated with the promotion of EMT. Thus, we have ascertained the relationship linking MSC and stemness and we found that, in melanoma cells, MSC medium enhanced key regulatory stem cell genes such as SOX2, c-Myc, Oct 3/4, and Nanog, and self-renewal ability. The SLC-0111 CAIX inhibitor was able to revert the potentiation of SOX2, c-Myc, Oct 3/4 and KFL4 and the self-renewal ability in MSC medium-exposed melanoma cells. CAIX activity inducing a reduced pH nearby stem cell microenvironment^40^, may lead, as suggested by Ledaki et al.^41^, to a “*stem cell pH buffer zone*”, e.g. an enriched low pH-protected zone of stem cells. We strong believe that a reduced pH is the best microenvironment in which stem cells might survive using diverse metabolic substrates and resist to any pro-apoptotic stress, including chemotherapy either for their acquired intrinsic ability or exerted by low pH itself^19^^,^^20^^,^^22^.

A final resistant aspect able to prevent a successful chemotherapy is the multi-drug resistant phenotype. It is now consolidated that, in cancer cells, a low pH favours the development of a drug resistant phenotype, and we have demonstrated that, in melanoma cells, extracellular microenvironment acidosis elicits Vemurafenib resistance^22^. Here we found that, in a BRAF^V600E^ mutated melanoma cells, MSC medium generates Vemurafenib resistant phenotype, as demonstrated by cell count, colony efficiency and level of expression of malignancy markers such as PDGF- and EGF-receptors, p-p70S6k and p-ERK/ERK ratio. On the other hand, when SLC-0111 CAIX inhibitor was used, MSC medium failed to sustain a Vemurafenib resistant phenotype and, in sensitised melanoma cells, Vemurafenib activity leads in to a down regulation of PDGF- EGF-receptors and p-p70S6k expression. Interestingly, EGFR and PDGFR have been demostrated to be critical drivers of the resistance to BRAF inhibitors in various type of cancers, including melanoma^25^^,^^42–45^.

The drug complementary activity of CAIX inhibitors is sustained by the finding of Federici *et al*.^46^ demonstrating a synergism of lansoprazole with CAIX inhibitors in treating melanoma cells. Further, Faes *et al*.^47^ revealed that inhibiting CAIX in different manner potentiates the activity of rapamycin against hypoxic human colorectal adenocarcinoma cells. The SLC-0111 Phase I clinical trial was just published allowing for the assessment of the dosage of the new drug to be used daily in cycles of 28 days. The study also showed few severe drug related adverse effects and recommended the Phase II dose for further clinical investigations^48^. In fact the Phase II clinical trials are currently proceeding both for pancreatic cancer and breast cancer^49^.

Our study reports a striking example of melanoma progression to a more malignant and resistant phenotype generated by MSC of tumour microenvironment and the possibility to contrast this diabolic liaison using CA inhibitors. Therefore, it is possible to believe that regardless of the wide resistant aspects expressed by melanoma cells exposed to MSC medium, all join on CAIX involvement: CAIX may well represent a target to treat resistant cancer cell subpopulations.

## Supplementary Material

Supplemental Material
