# Supplementary material for: The Carbonic Anhydrase IX inhibitor SLC-0111 as emerging agent against the mesenchymal stem cell-derived pro-survival effects on melanoma cells
Source: J Enzyme Inhib Med Chem. 2020 May 12;35(1):1185–93. doi: 10.1080/14756366.2020.1764549 (PMC7269050; doi:10.1080/14756366.2020.1764549)

Figure S1: CAIX expression on MSC. MSC were stained with CAIX- specific antibody and analyzed with cytometer. Numbers indicate the % of stained cells.

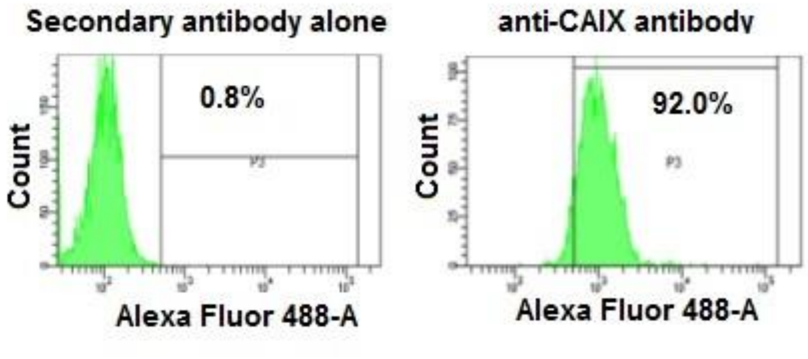

Supplement: Supplemental Material [file IENZ_A_1764549_SM3076.pdf]
